# The structure of a prophenoloxidase (PPO) from *Anopheles gambiae* provides new insights into the mechanism of PPO activation

**DOI:** 10.1186/s12915-015-0225-2

**Published:** 2016-01-05

**Authors:** Yingxia Hu, Yang Wang, Junpeng Deng, Haobo Jiang

**Affiliations:** Department of Biochemistry and Molecular Biology, Oklahoma State University, Stillwater, OK 74078 USA; Department of Entomology and Plant Pathology, Oklahoma State University, Stillwater, OK 74078 USA

**Keywords:** Innate immunity, Melanization, Mosquito, Type III copper proteins, Zymogen activation

## Abstract

**Background:**

Phenoloxidase (PO)-catalyzed melanization is a universal defense mechanism of insects against pathogenic and parasitic infections. In mosquitos such as *Anopheles gambiae*, melanotic encapsulation is a resistance mechanism against certain parasites that cause malaria and filariasis. PO is initially synthesized by hemocytes and released into hemolymph as inactive prophenoloxidase (PPO), which is activated by a serine protease cascade upon recognition of foreign invaders. The mechanisms of PPO activation and PO catalysis have been elusive.

**Results:**

Herein, we report the crystal structure of PPO8 from *A. gambiae* at 2.6 Å resolution. PPO8 forms a homodimer with each subunit displaying a classical type III di-copper active center. Our molecular docking and mutagenesis studies revealed a new substrate-binding site with Glu364 as the catalytic residue responsible for the deprotonation of mono- and di-phenolic substrates. Mutation of Glu364 severely impaired both the monophenol hydroxylase and diphenoloxidase activities of AgPPO8. Our data suggested that the newly identified substrate-binding pocket is the actual site for catalysis, and PPO activation could be achieved without withdrawing the conserved phenylalanine residue that was previously deemed as the substrate ‘placeholder’.

**Conclusions:**

We present the structural and functional data from a mosquito PPO. Our results revealed a novel substrate-binding site with Glu364 identified as the key catalytic residue for PO enzymatic activities. Our data offered a new model for PPO activation at the molecular level, which differs from the canonical mechanism that demands withdrawing a blocking phenylalanine residue from the previously deemed substrate-binding site. This study provides new insights into the mechanisms of PPO activation and enzymatic catalysis of PO.

**Electronic supplementary material:**

The online version of this article (doi:10.1186/s12915-015-0225-2) contains supplementary material, which is available to authorized users.

## Background

Phenoloxidase (PO) is a critical enzyme involved in multiple physiological processes including innate immunity of insects and crustaceans. As the close homolog of arthropod hemocyanins, PO is produced in hemocytes as a zymogen, the prophenoloxidase (PPO) [1]. Upon pathogenic infections or physical injuries, a serine protease cascade is triggered to activate PPO as a local response. The last step of PPO activation involves a trypsin-like enzyme, named PPO-activating proteinase (PAP), which cleaves PPO at a conserved proteolytic cleavage site near the N-terminus to generate active PO [2, 3]. In vitro, PPO can also be activated without proteolytic cleavage by certain chemicals such as ethanol or detergents (e.g. cetylpyridinium chloride, CPC) [4–6].

In general, the active PO possesses *o*-hydroxylase (EC 1.14.18.1) and *o*-diphenoloxidase (EC 1.10.3.1) activities that convert a variety of monophenolic and *o*-diphenolic substrates to *o*-quinones [6, 7]. Quinones may act as cross-linkers for wound healing, and they also polymerize to form melanin capsules around parasites and parasitoids [7, 8]. Quinones and other reactive intermediates (e.g. 5,6-dihydroxyindole) directly kill microbial pathogens and parasitoids [9].

POs, together with hemocyanins, tyrosinases, and catechol oxidases, belong to the type III di-copper family of proteins, which share an antiferromagnetically coupled di-copper center [10, 11]. This group of proteins are widely distributed in different organisms: POs in arthropods, tyrosinases in microbes, plants and mammals, catechol oxidases in plants and fungi, and hemocyanins in arthropods and molluscs [12–14]. Tyrosinases and catechol oxidases are responsible for browning of fruits and plants, and they may also play a role in defense mechanism in plants and fungi [15]. Mammalian tyrosinase is a major enzyme required for coloring of hair, skin and eyes, and its deficiency and excessive expression could lead to albinism and skin cancer, respectively [7, 16, 17]. Hemocyanins were initially recognized as oxygen carriers in hemolymph [18], but their PO activities and roles in antimicrobial defense were discovered later [6, 19]. In spite of having a similar active site, vital structural and functional differences do exist in these proteins as has been demonstrated in the past decades. Tyrosinases and POs catalyze both the *o*-hydroxylation and oxidation reactions, but catechol oxidases only possess the oxidase activity [20, 21]. Based on their similarities in the primary and tertiary structures, POs were considered to be evolutionarily more related to arthropod hemocyanins, whereas tyrosinases and catechol oxidases are closer to molluscan hemocyanins [19, 22, 23].

In the catalytic cycle, type III di-copper center goes through three redox states: the reduced deoxy state [Cu(I)-Cu(I)], the oxy state [Cu(II)-O_2_^2−^-Cu(II)] in which a peroxide molecule binds to the two Cu ions in a μ-η^2^:η^2^ side-on bridging fashion, and the met state [Cu(II)-OH^−^-Cu(II)] in which the two Cu ions are ligated to a hydroxide ion [10]. The oxy state enzyme is capable of catalyzing both the hydroxylation of monophenols and the oxidation of *o*-diphenols to *o*-quinones, while the met state only undertakes the latter diphenol oxidase reaction [10].

PO catalyzed-melanogenesis is indispensable in the immune system of invertebrates [24, 25] and two PPO structures from *Manduca sexta* and *Marsupenaeus japonicus* have been investigated [26, 27]. Although these studies on PPO provided important structural and functional insights, the detailed enzymatic mechanism of PO is still undetermined and the fundamentals of PPO activation remain elusive. *A. gambiae* is a major vector of human malaria parasites in Africa, whose innate immune system protects the mosquito from infection by incompatible malaria parasites [28]. It contains nine PPO genes which are expressed at different tissues and life stages [29–32]. Herein, we report the crystal structure of AgPPO8 at 2.6 Å resolution, representing the first structure from a recombinant PPO and the first from a mosquito species. Our structural and functional studies on AgPPO8 revealed a novel substrate-binding pocket that differs from the previously deemed ‘placeholder’ position occupied by a phenylalanine residue, which is conserved in PPOs, but not in molluscan hemocyanins, tyrosinases, or catechol oxidases. We identified E364 as a catalytic residue key to the PO activities. Our data provide new insights into the mechanism of PO catalysis, which could be applicable to other type III di-copper proteins, and suggest a new model for PPO activation at the molecular level.

## Results

### Overall structure of AgPPO8

**Fig. 1:**
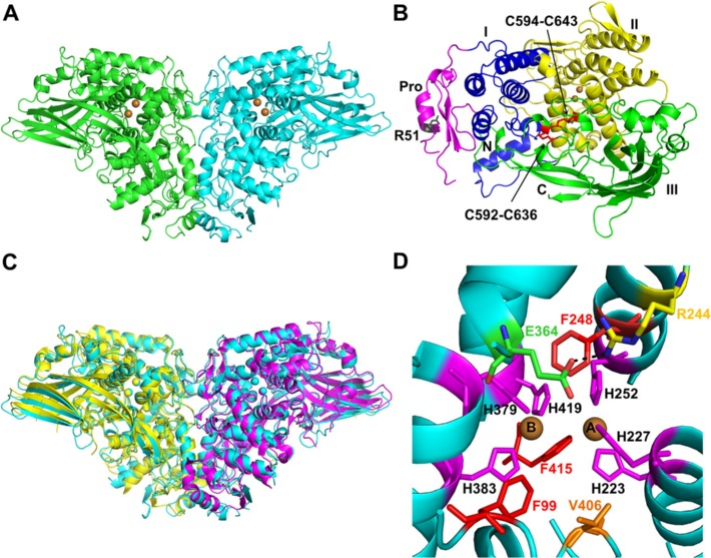
Crystal structure of *A. gambiae* PPO8. (**a**) Overall structure of the homodimer with each subunit shown in cyan and green, respectively. Cu atoms are shown as brown spheres. (**b**) The domain structure of AgPPO8: pro-region, magenta; domain I, blue; domain II, yellow; domain III, green. The Cu atoms are shown as brown spheres and the disulfide bonds are shown as red sticks. The side chain of Arg51 preceding the proteolytic cleavage site is shown as a black stick. (**c**) Superposition of AgPPO8 (cyan) with *M. sexta* PPO heterodimer (PPO-1 in magenta and 2 in yellow, PDB ID 3HHS). AgPPO8 is 41.1 % identical in a.a. sequence to MsPPO1 and 38.8 % to MsPPO2. (**d**) The di-copper active site of *A. gambiae* PPO8. The Cu atoms are shown as brown spheres. Six conserved His residues (magenta) coordinate CuA and CuB, and are stabilized by three Phe residues (F248, F415 and the 'placeholder' F99, shown as red sticks). V406 (orange) aligns to a putative catalytic residue E395 in MsPPO2. Glu364 (green) forms a salt bridge (black dash) with R244 (yellow), and is required for the enzymatic activities of AgPPO8. The secondary structures of AgPPO8 are shown and colored in cyan

The crystal structure of AgPPO8 contains two identical subunits, each comprising 700 residues, which are associated tightly to form a homodimer in the asymmetric unit. The overall structure of the AgPPO8 displays a butterfly shape of  147 × 70 × 60 Å in size (Fig. 1a). Most residues are well defined, except for 40 amino acids that lack electron densities. These include residues 59–65, 580–587, 629–632, and 698–700 in chain A and residues 1, 2, 65–68, 579–587, and 698–700 in chain B. AgPPO8 adopts a similar architecture as that of *M. sexta* PPO (MsPPO) [26], with the three conserved domains (Fig. 1b). The pro-region (17–81) contains the conserved proteolytic cleavage site (R51*F52). Domain I (1–16, 82–196) is dominant in α-helices. Domain II (197–435) contains the di-copper active site buried in an α-helix bundle. Domain III (436–697) is mainly a seven-stranded β barrel, with an additional β-hairpin extended to domains I and II. There are two disulfide bonds (C592-C636 and C594-C643) within domain III of each subunit, which are highly conserved among hemocyanins and PPOs. C592-C636 is situated on the surface and exposed to the solvent, while C594-C643 is located at the interface of domains II and III, which may contribute to the structural stability. The overall structure of AgPPO8 homodimer closely resembles that of MsPPO heterodimer (Fig. 1c), with a 1.14 Å root-mean-square deviation over 1,129 aligned Cα atoms.

### Dimerization of AgPPO8

AgPPO8 was found to stay mainly as a homodimer in solution by size exclusion chromatography and dynamic light scattering (Additional file 1). Two types of dimerization patterns exist in the crystal structure, a tight homodimer and a loose one. The tight dimer associates (chain A and chain B) via a two-fold non-crystallographic symmetry axis in the asymmetric unit. The loose dimer involves chain A molecule and a crystallographic symmetry-related chain B molecule in the crystal lattice. The tight dimer interface is mainly stabilized through extensive hydrophobic and charge-charge interactions predominantly from domains I and II, burying a large (4,624 Å solvent accessible surface area which is comparable to that of MsPPO heterodimer [26]. In contrast, the loose dimer buries a much smaller surface area ( 934 Å^2^) at the interface. The tight homo-dimeric association of AgPPO8 was also confirmed with an analysis performed with the PISA server (http://www.ebi.ac.uk/msd-srv/prot_int/pistart.html), suggesting it is the biologically functional dimer.

### Active site of AgPPO8

During refinement, we observed a large positive difference electron density at the supposed bi-metal center (Additional file 2), although no additional metal ions were supplied during the protein expression. We believe the bound metal ions came from the trace amount of metal in LB medium. The identities of the metal consequently could not be unambiguously determined in this study, since either Zn or Cu atoms could be positioned at the same locations and refine well. This observation is consistent with a previous report that heterogeneous incorporation of zinc and copper ions was found at the active site of *Bacillus megaterium* tyrosinase [33]. In this study, we interpreted the metals as Cu ions and finalized that in the deposited structure. Each metal ion is coordinated with the NE2 atoms of three histidine residues, which are highly conserved in this protein family (Fig. 1d): CuA is associated with H223, H227 and H252, while CuB is associated with H379, H383 and H419. All six histidine residues are located in the α-helix bundle from domain II, and stabilized by three Phe residues (F99, F248 and F415) through hydrophobic interactions: F99 with H379 and H383, F248 with H227, H252 and H379, and F415 with H223, H252, H383 and H419 (Additional file 3). The distance between the two copper atoms is 4.5 Å, which is the typical distance for the reduced form of type III di-copper proteins. The UV/Vis absorption spectrum did not exhibit any apparent characteristic feature from 250 to 700 nm (Additional file 4), supporting that the AgPPO8 structure we obtained was in the deoxy state.

**Fig. 2:**
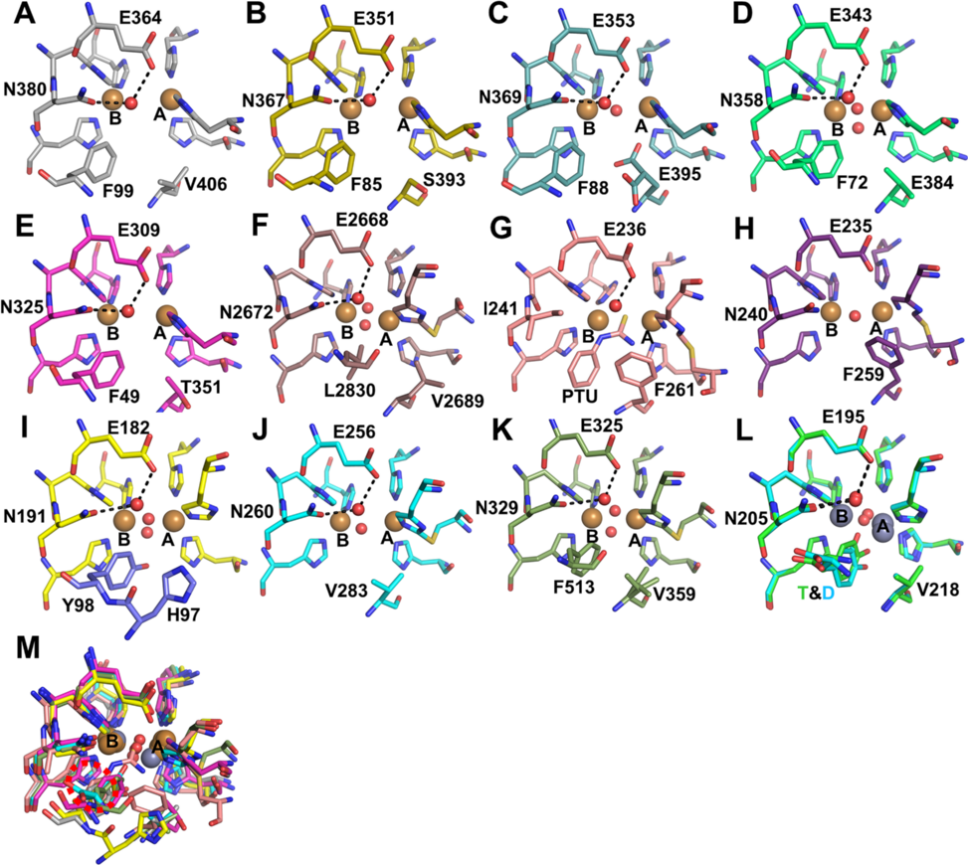
Comparison of the di-copper centers from different type III copper proteins. (**a**) *A. gambiae* PPO8 (AgPPO8), PDB ID 4YZW; (**b**) *M. sexta* PPO1 (MsPPO1), 3HHS-B; (**c**) *M. sexta* PPO2 (MsPPO2), 3HHS-A; (**d**) *Marsupenaeus japonicus* (Mj) PPOβ, 3WKY; (**e**) *Limulus polyphemus* hemocyanin deoxy state (LpHc), 1LLA; (**f**) *Octopus* hemocyanin functional unit Odg, 1JS8; (**g**) *Ipomoea batatas* catechol oxidase (IbCO) in complex with 1-phenyl-2-thiourea (PTU), 1BUG; (**h**) *Vitis vinifera* catechol oxidase (VvCO), 2P3X; (**i**) *Streptomyces castaneoglobisporus* tyrosinase (ScTyr), 2ZMX; (**j**) *Agaricus bisporus* tyrosinase (AbTyr), 2Y9W; (**k**) *Aspergillus oryzae* pro-tyrosinase (AoProTyr), 3W6W; (**l**) Superposition of BmTyr structures in complex with bound tyrosine (4P6R) and *L*-DOPA (4P6S) (BmTyr:T/D); (**m**) Superposition of the active sites from AgPPO8, ScTyr, AoProTyr, LpHc and BmTyr:D. Space overlapping between the placeholders and phenolic substrate is indicated by a red dashed cycle. The Cu or Zn atoms are shown as large brown or gray spheres, the Cu-coordinated or hydrogen-bonded water molecules are shown as small red spheres. The hydrogen bonds are indicated as black dashes

The di-copper center observed in AgPPO8 is similar to those in other type III copper enzymes. However, the catalytic residue has been elusive due to the amino acid variations near the dicopper center (Fig. 2). In MsPPO2, one unique acidic residue (E395) was proposed to be a catalytic residue responsible for the hydroxylase activity of the enzyme since its carboxylic oxygen in the side chain may deprotonate the hydroxyl group of the monophenol substrate (Fig. 2c) [26, 34]. Remarkably, although AgPPO8 was able to convert monophenol to diphenol (see below), it contains a valine residue (V406) at the equivalent position, which lacks an acidic side chain (Fig. 1d, 2a). Therefore, the deprotonation of the phenolic substrate during AgPPO8 enzymatic catalysis may involve a different residue.

### Substrate binding by AgPPO8

**Fig. 3:**
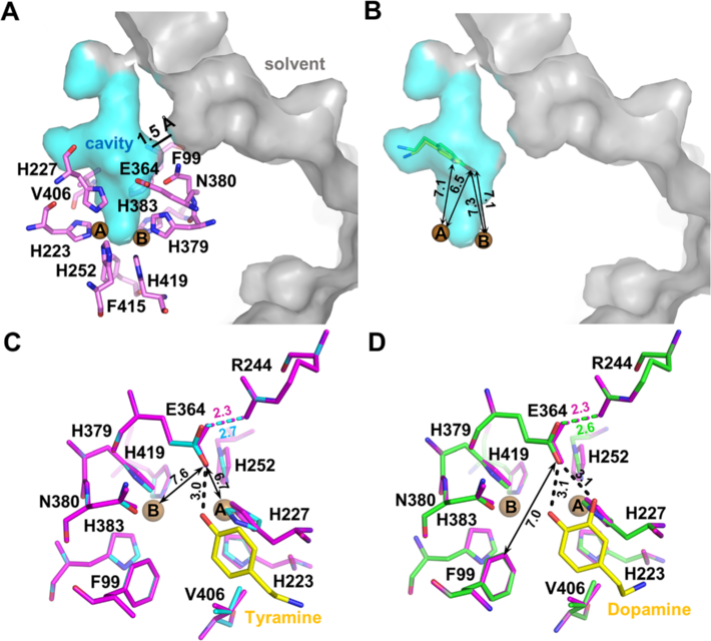
A new and unique substrate-binding pocket (Site II) of AgPPO8. (**a**) AgPPO8 contains a cavity approximate to surface (Site II, side view). The cavity and solvent exposed surface areas are represented in cyan and gray, respectively. The key residues surrounding the cavity are shown as violet sticks. (**b**) The side view of Site II pocket with tyramine (green) and dopamine (yellow) docked inside. The distances (Å) from the phenolic oxygens of the substrates to CuA and CuB are indicated. (**c** and **d**) Superposition of the active sites before and after docking. The active site residues of the apo-AgPPO8 are shown as magenta sticks, while the complex structures with docked substrates bound are colored in cyan (tyramine) and green (dopamine), respectively. The hydrogen bonding between the substrate and E364 is highlighted with black dash. The distances (Å) from E364 to CuA, CuB, the putative placeholder F99, and the phenolic oxygens of docked substrates (yellow sticks) are indicated. The distance of the salt bridge between residues E364 and R244 is also labeled (before docking, magenta; after docking, tyramine, cyan and dopamine, green). CuA and CuB are shown as brown spheres

As seen in other arthropod PPOs and hemocyanins, AgPPO8 also contains a putative ‘placeholder’ for phenolic substrates, which is F99 in each subunit (Fig. 1d, Additional file 3). This conserved residue resides in a loop of domain I with its aromatic side chain protruding to the di-copper center and stacking onto the imidazole ring of H383 at the CuB site. It was assumed in the prior studies that the placeholder aromatic residue would be withdrawn from the di-copper center during PPO activation through major conformational changes (see discussion), allowing its position be replaced by a natural phenolic substrate during enzymatic catalysis. Interestingly, the analysis of the AgPPO8 structure revealed a prominent cavity in the di-copper reaction center, which is close (~1.5 Å) to the solvent-accessible surface (Fig. 3a). This cavity is surrounded by the six Cu-coordinating His residues, F99, F415, E364, N380, V406, along with other residues from domain I and II (Fig. 3a). The volume of the cavity (451 Å^3^, calculated by program CASTp [35]) appears large enough to accommodate monophenolic or diphenolic substrates such as tyramine and dopamine (approximately 190–200 Å^3^). Therefore, we named this pocket as the second substrate-binding site (Site II) with respect to the placeholder position (Site I). To test if this pocket could hold substrates, we carried out docking experiments using the Autodock program. After selecting certain amino acids at the active site as the flexible residues to accommodate potential protein dynamics (see Methods), we individually docked tyramine and dopamine into AgPPO8 (Additional file 5). Although a larger grid box covering the entire di-copper center and the empty cavity was set as the search space, both substrates were successfully docked into the putative substrate-binding Site II in similar orientations (Fig. 3b) without significant reconfiguration of the active site (Fig. 3c,d). This observation suggested substrate binding to PPO could be achieved without displacing the placeholder. Interestingly, in the docked protein structures, we found residue E364 located very close to the docked substrates (Fig. 3c,d). Albeit distant from the di-copper center (6.7 and 7.6 Å from CuA and CuB in apo-AgPPO8), the OE2 atom of E364 is 3.0 and 3.1 Å to the phenolic oxygens of tyramine and dopamine, respectively. This observation suggested that E364 could play an important role in the deprotonation of the phenolic substrates, which was shown to be essential for binding of the phenolic oxygen to one of the copper atoms [34, 36, 37]. E364 also forms a salt bridge with R244 in a stable conformation, which may assist in correctly positioning the carboxylic side chain during the deprotonation process (Figs. 1d and 3c,d). We found this Glu residue highly conserved among other type III di-copper proteins (Fig. 2). These observations indicated that this Glu residue could be a common catalytic residue for PO enzymatic activities, and may serve in an alternative mechanism for PPO activation (see below).

### E364 is key to hydroxylation and oxidation

**Fig. 4:**
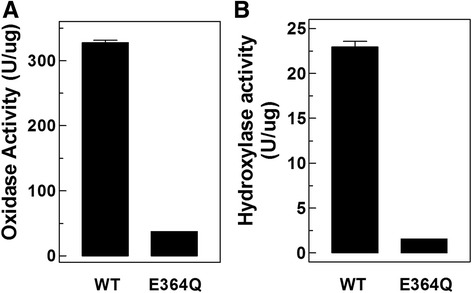
E364 is key to PO enzymatic catalysis. (**a**) E364Q mutation severely impairs diphenol oxidase activity of AgPPO8. Dopamine was used as the substrate and specific PO activity was shown as ΔmOD_470_/min/μg or U/μg; *P* <0.001. (**b**) E364Q mutation severely impairs the monophenol hydroxylase activity of AgPPO8. Tyramine was used as the substrate and the rate of dopamine formation catalyzed by 1 μg enzyme was estimated as ΔmOD_280_/min/μg or U/μg; *P* <0.001. All data were presented as mean ± SEM (n = 3)

To test our hypothesis that E364 is essential for the enzymatic activities of AgPPO8 by deprotonating phenolic substrates, we mutated it to a neutral Gln residue. The recombinant E364Q mutant was expressed as a soluble protein in *E. coli* cells and folded properly with an approaching melting point as the wild type as shown from the Differential Scanning Fluorimetry analysis (Additional file 6). The enzymatic activities of both wild type and mutant AgPPO8 were assayed in vitro*.* When using dopamine as the substrate, the E364Q mutant lost nearly 89 % of its oxidase activity compared to the wild type (Fig. 4a), indicating E364 is the base responsible for the deprotonation of diphenolic substrate. When using tyramine as the substrate, E364Q mutant impaired 93 % of its hydroxylase activity compared to the wild type (Fig. 4b), by detecting the dopamine formation at 280 nm. This demonstrated that E364 is also necessary for deprotonating the monophenolic substrate. Taken together, we conclude that E364 is essential for both the hydroxylase and oxidase activities of AgPPO8 with its carboxylate group acting as a general base for monophenol and diphenol deprotonation.

## Discussion

In this study, we determined the crystal structure of AgPPO8, which is the first structure of recombinant PPO and the first from a mosquito species. The structural and functional studies on AgPPO8 revealed a novel substrate-binding pocket and identified E364 as a catalytic residue key to the PO activities. Our data also provide new insights into the catalytic mechanism of PO and suggest a new model for PPO activation at the molecular level.

### Catalytic residues for phenol deprotonation

Enzymatic activities of type III copper protein require deprotonation of the phenolic substrates, which is essential for coordinating the phenolic oxygen to one of the copper atoms and subsequent catalytic reactions [34, 36, 37]. So far, at least five different residues have been proposed as the potential deprotonating base. In *Streptomyces castaneoglobisporus* tyrosinase (ScTyr), a flexible, CuA-coordinating H54 was supposed to serve as the base [38]. This is not applicable to PPOs due to inflexibility of their His residues in the active site [26]. In mouse tyrosinase, a free His preceding the sixth coordinating His is vital for the oxidation of diphenols [39], yet this residue is absent in arthropod hemocyanins and PPOs. In MsPPO2, there are two acidic Glu residues in the vicinity of its binuclear center, E353 (equivalent to E364 of AgPPO8) and E395 [26]. Since E395 is located much closer to CuA, the presumed site for hydroxylase activity [40, 41], and within 3 Å distance from residue F88, the ‘placeholder’ for phenolic substrates, it was postulated to be responsible for tyrosine deprotonation in the monooxygenase reaction. However, this Glu residue is not present in most type III di-copper proteins that exhibit the hydroxylase activity (Fig. 2). Due to technical difficulties in expressing recombinant MsPPOs, the putative catalytic function of E395 in MsPPO2 has not been verified experimentally through mutagenesis analysis. Recent studies on *B. megaterium* tyrosinase provided complex structures in met form with bound tyrosine or *L*-DOPA (BmTyr:T/D), revealing that the CuA site is solely responsible for its monooxygenase and diphenoloxidase activities [42]. A conserved water molecule activated by E195 was proposed to be the intermediate base for deprotonating the entering substrates, since it is closer to the di-copper center than the candidate residue E195 (Fig. 2l). Nevertheless, the water molecule was not observed to have direct contact with the bound substrates in the met form structures. In AgPPO8, E364 is the equivalent residue to BmTyr E195. The proposed catalytic water molecule also exists in the current structure, which is situated at a 6 Å distance from CuA and stabilized by E364 and N380 (Fig. 2a). It may be possible that AgPPO8 also adopts a similar water-mediated deprotonation mechanism in its catalytic reaction. However, the role of this water molecule has not been confirmed since it is not found in the crystal structure of *Vitis vinifera* catechol oxidase (VvCO) (Fig. 2h), which contains the conserved E235 and N240 [43]. In the structure of *Ipomoea batatas* catechol oxidase (IbCO) (Fig. 2g), E236 was suggested to be responsible for the deprotonation of diphenolic substrates [44]. This Glu also aligns to E364 of AgPPO8, which is in fact highly conserved among type III di-copper proteins (Fig. 2). Our functional study confirmed the importance of E364 in both hydroxylase and oxidase activities of AgPPO8, representing the crucial mutagenesis data for PPO for the first time in the field. Collectively, these lines of evidence suggest that the Glu residue at this position is essential to type III copper enzyme activities by playing a critical role in the deprotonation of phenolic substrates. E364 of AgPPO8 is 7 Å away from the putative ‘placeholder’ F99 (Fig. 3d), making it unlikely to deprotonate the phenolic substrate at Site I. In contrast, E364 is in proximity to the docked substrates in the newly identified substrate-binding pocket Site II, implying that Site II might be the actual substrate-binding site for PO activities, at least for the initial substrate binding, which is independent of the canonical ‘placeholder’ position.

### The activation mechanism of PPOs: a loop gated entrance for substrate

PPO is synthesized in hemocytes and released to plasma, while partially transported to cuticles [45]. The activation of MsPPO requires the presence of PAP and SPHs simultaneously [46]. Without the SPHs, PAP can cleave PPO at the correct position but the product does not display PO activities. It was hypothesized that PPO activation is carried out by a PAP on the surface of a large complex of the SPHs [47]. In vitro, it was shown that PPO could be alternatively activated by treatment of detergents without proteolytic cleavage [4]. However, the mechanism of PPO activation remains elusive at the molecular level.

The di-copper active centers of all known type-3 copper protein structures could be well superimposed (Fig. 2). It was supposed that, in these structures, the entrances to the di-copper center are blocked by hydrophobic residues that need to be dislodged for activation. The blocker is a highly conserved Phe residue in all known arthropod hemocyanins, and a less conserved aliphatic amino acid in mollusc hemocyanins [11, 19]. In ScTyr, a caddie protein ORF378 acts as a shielding domain with its Y98 inserted into the substrate-binding pocket of tyrosinase [38]. This Tyr residue is kept away from the active site by the caddie protein at a sufficient distance to avoid reaction. The structure of *Aspergillus oryzae* pro-tyrosinase (AoProTyr) also confirms that F513 of C-terminal domain extends into the binuclear active site, protecting the enzyme from early reaction [48]. In the structure of IbCO, an inhibitor (1-phenyl-2-thiourea) is located at the equivalent position [44]. Because the aromatic rings of these blocking residues could be well superimposed to each other, they were suggested as the placeholders for incoming substrates and are stabilized by stacking interactions with a His residue at the CuB site [40]. By overlaying the active sites of these known type III protein structures onto the BmTyr:T/D complex [42], space clashes between the placeholders and phenolic substrates can be easily detected (Fig. 2m). These data implied that the placeholder must be removed to accommodate the substrate binding. In arthropod PPOs, a conserved Phe residue from the N-terminal domain I was considered as the substrate placeholder [26, 27]. Therefore, dislocation of the placeholder to make place for substrate access was assumed as a necessary step for PPO activation.

Hemocyanins are generally functional as oxygen carriers, although they were shown to be able to get activated in vitro by certain detergents and chemicals in the same way as PPOs [6, 19]. The conformational changes of the placeholder Phe residue in certain hemocyanins were observed. For example, the structural comparison of *L. polyphemus* hemocyanin subunit II of oxygenated state with deoxygenated *Panulirus interruptus* hemocyanin revealed an 8° rotation of domain I upon oxygen binding, pulling the placeholder F49 away from the active site [49, 50]. SDS activation of *P. imperator* hemocyanin oligomer also twisted domain I away from domains II and III, consequently removing F49 about 3.5 Å away from its original position [51].

**Fig. 5:**
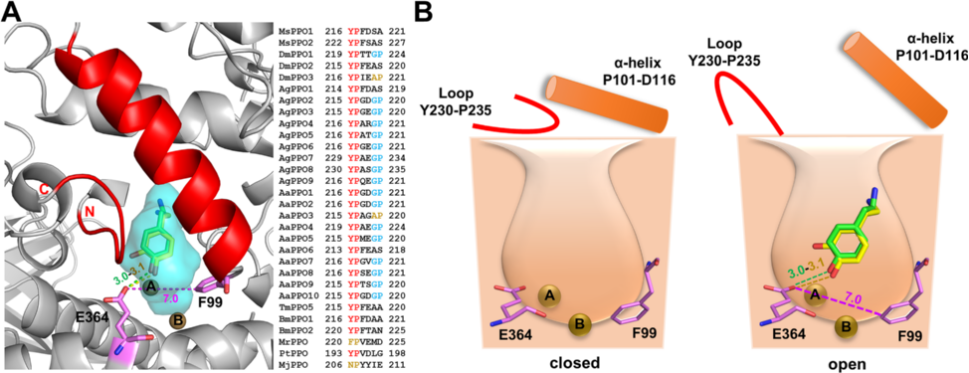
A loop gating model for AgPPO8 activation. (**a**) The top view of Site II pocket with docked tyramine (green) and dopamine (yellow). The secondary structures of AgPPO8 are shown in gray. The flexible loop Y230-P235 (red) forms loose van der Waals contacts with the α-helix P101-D116 (red), controlling the entrance to the substrate binding Site II (cyan). The putative placeholder F99 and the catalytic residue E364 are shown as violet sticks. Inset shows the sequence alignment of the loop among different PPOs from insects and crustaceans. Ms, *Manduca sexta*; Dm, *Drosophila melanogaster*; Ag, *Anopheles gambiae*; Aa, *Aedes aegypti*; Tm, *Tenebrio molitor*; Bm, *Bombyx mori*; Mr, *Macrobrachium rosenbergii*; Pt, *Portunus trituberculatus*; Mj, *Marsupenaeus japonicas*. The distances from E364 to the putative placeholder F99 (magenta) and the docked tyramine (green)/dopamine (yellow) are indicated. (**b**) A cartoon illustration of the loop gated AgPPO8 activation. The side view of the Site II pocket is shown with the loop-helix controlled entrance located at the top. Left, closed state. Right, open state. Conformational changes at the molecular surface disrupt the loose contact between the loop Y230-P235 and the helix P101-D116, opening the gate and allowing the substrates to access the active site

The structure of AgPPO8 revealed an alternative substrate-binding Site II. In our docking analysis of AgPPO8, substrates could be accommodated in the putative binding pocket Site II without displacing the placeholder F99. This observation led to a reevaluation of the activation mechanism of type III di-copper proteins. There is a profound structural difference between the proteolytic activation of PPOs and other precursors of type III di-copper proteins. In pro-tyrosinases and hemocyanins, the proteolysis removes the shielding domain including the placeholder, exposing the catalytic di-copper center for substrate binding [48, 52]. A similar activation mechanism was also suggested for pro-catechol oxidases [53], while in PPOs multiple cleavage sites have been identified among different species [13, 46, 54–59]. In most cases, the proteolytic cleavage of the N-terminal fragment does not remove the placeholder, which remains in the core of the enzyme downstream to the proteolytic cleavage site [46, 57–59], and a subsequent conformational change is required to induce the enzyme activity since the active site is still buried. Indeed, the conserved placeholder Phe residue of PPOs is involved in extensive non-polar interactions with the Cu-coordinating His residues, stabilizing the active center. Dislodging the placeholder in PPO would require major conformational changes involving disruption of multiple domain–domain interactions. Should this happen in vivo, it could possibly be assisted by other protein complexes, which stabilize the labile intermediate conformation. However, a smaller conformational change could be sufficient for substrate to enter the active center via Site II (Fig. 5). The putative substrate-binding pocket in AgPPO8 is close to the solvent with the entrance gated by a flexible loop YPASGP (230–235) from domain II and the α-helix P101-D116 from domain I at the molecular surface (Fig. 5a). The loop contacts the α-helix merely via loose van der Waals interactions, suggesting that reorientation of the loop by modulating PPO surface plasticity is possible. The N-terminal Tyr-Pro and C-terminal Gly-Pro of the loop, which are highly conserved among different PPOs (Fig. 5a, Inset), have a higher frequency of occurrences in *cis*-*trans* conformational switching [60, 61], and may therefore serve as the hinge facilitating the loop to flip over and open up the gate towards Site II, allowing its access by the substrates (Fig. 5b). This model apparently does not need the withdrawal of the placeholder F99, which is buried in the core of the protein. Notably, when we manually removed the placeholder F99 from the structure of AgPPO8 in the docking experiment, the phenolic substrate still docked to Site II (Additional file 7), further supporting its preferential binding of substrate. Taking into account that the phenolic oxygen of the docked substrates is 6.5–7.3 Å away from the di-nuclear coppers (Fig. 3b), the orientation of docked substrates could represent the initial substrate binding. Further shifting of the substrate towards the di-copper center is required in order for the catalysis to occur. In fact, there is extra vacant space within the Site II pocket immediately above the di-copper center (Fig. 3b). Therefore, subsequent fine-tuning of the active site geometry could be sufficient for coordinating the bound substrate to the di-copper atoms within Site II pocket.

Our data here suggested an alternative model for PPO activation without displacing the canonical ‘placeholder’ Phe residue. This model implies that Site II could be the actual substrate-binding site for PO activities. The PPO activation process involves a movement of a flexible loop at the entrance to the substrate binding Site II. This conformation change could be induced by the interaction of PPO with other molecules. In vitro, the binding of ionic detergents to the charged surface of PPO could induce allosteric conformational changes at the loop Y230-P235. In vivo, the mechanism of PPO activation is not yet well understood, but our data presented here suggest that a similar activation mechanism could also be adopted. The opening of the gate at Site II could be modulated by exquisite protein–protein interactions between PPO and the activating protein complexes, which have been extensively investigated in the prior studies [47, 62–65]. It is possible that the proteolytic cleavage of PPO in the pro-region could simply create surface complementarities between these proteins for optimal binding and recruitment [26]. The ‘placeholder’ Phe residue therefore may not be dislodged from its original stable position during this PPO activation process.

## Conclusions

In this work, we determined the crystal structure of a recombinant PPO8 from a mosquito species, *Anopheles gambiae*, which forms a homodimer with each subunit containing a conserved type III di-copper active site. We identified E364 as the catalytic residue key to the PO activities through mutagenesis and functional analysis, which elucidated a conserved catalytic mechanism applicable to other type III di-copper enzymes. Our results also revealed the actual substrate-binding site and offered a novel model for PPO activation without withdrawing the previously proposed ‘placeholder’ phenylalanine residue. The data we presented here provides new insights into the mechanisms of PPO activation and enzymatic catalysis, and should provide an important focus for future investigations.

## Methods

### Plasmid construction, protein expression, purification and crystallization

The DNA of the 5’ and 3’ AgPPO8 cDNA fragments were amplified from a cDNA pool of second instar *A. gambiae* larvae. The PCR products were separately ligated with pGEM-T DNA (Promega) for transforming JM109 (Promega). After sequence verification, the 5’ and 3’ fragments were retrieved and ligated with a modified pET vector as a SUMO fusion with an N-terminal 6xHis-tag. There are two additional FLAG and c-Myc tags next to the N-terminal and C-terminal of AgPPO8 sequence, respectively, which are used for functional studies other than this work. Protein expression was carried out in *E. coli* BL21 gold (DE3) cells (Stratagene). The colony carrying the recombinant plasmid was grown in LB medium and protein expression was induced by 0.5 mM isopropyl-β-D-thiogalactopyranoside at 16 °C for 16 h. The point mutation of AgPPO8 was constructed following the protocol of QuikChange II Site-Directed Mutagenesis Kit (Agilent Technologies, Inc.). The AgPPO8 mutant was expressed in the same way as the wild type protein. The individual proteins were purified using a similar double Ni-nitrilotriacetic acid procedure as previously described [66]. The purified AgPPO8 was concentrated to 7.2 mg/mL. For optimal reproducibility of crystallization, all purified proteins were flash frozen and stored at −80 °C until usage [67]. AgPPO8 crystallized in sitting drops at room temperature with a reservoir solution containing 0.2 M lithium citrate tribasic tetrahydrate, 20 % PEG 3,350, at pH 8.4. Crystals were cryoprotected by soaking in mother crystallization solution containing 20 % glycerol.

### Data collection and structural determination

A set of data was collected at 100 K to 2.60 Å resolution at the Advanced Photon Source, beam-line 19-ID, Argonne National Laboratory (Argonne, IL) and processed by the HKL3000 program [68]. The initial phasing was obtained by molecular replacement with the Phaser program of CCP4 suit, in which chain B of the crystal structure of MsPPOs (PDB code 3HHS) was used as the searching model. Subsequent model building was carried out by Autobuild program of Phenix [69] coupled with manual modeling using WinCoot [70]. The structure was further refined using Phenix.refine and the final model was analyzed by the Molprobity server [71]. The current model is of excellent geometry and refinement statistics (Table 1), and validated by wwpdb validation servers [72]. The structure factors and atomic coordinates for AgPPO8 have been deposited in the protein data bank with accession code 4YZW. All structural figures were generated using PyMol [73].

**Table 1:** Data collection and refinement statistics

Crystal data	
Beam-line	19-ID APS
Wavelength, Å	0.97915
Space group	P 2_1_
Cell constants	a = 75.6 Å, b = 106.6 Å, c = 92.1 Å, β = 105.8°
Resolution, Å	2.60
Total reflections	214,320
Unique reflections	43,019
Completeness, %	99.0 (97.3)
I/σ	8.9 (1.8)
R_sym_, %	12.9 (47.2)
Refinement statistics	
Reflection range used, Å	2.60–44.32
No. reflections used	42,950
R_work_/R_free_, %	18.8/23.3
rmsd bonds, Å	0.0046
rmsd angle, °	0.791
Ramachandran plot (preferred/allowed), %	96.6/3.4
No. of atoms	
Protein	10,901
Metal ions	4
Waters	263

*R*_*sym*_ = ∑|*I*_obs_ - *I*_avg_|/ ∑ *I*_avg_; R_work_ = ∑ ││*F*_obs_ – *F*_calc_││/ ∑ *F*_obs_

*R*_*free*_ was calculated using 5 % data

APS, Advanced Photon Source; I/σ, Intensity/Sigma (Intensity). Values in parentheses are for the highest-resolution shell 2.69–2.60 Å

### UV/vis absorption spectrophotometry

Beckman DU520 General Purpose UV/vis Spectrophotometer was used for recording the UV/vis absorption spectrum of AgPPO8. A quartz micro cell cuvette of 1 cm path length was used. AgPPO8 protein was diluted to 0.5 mg/mL, with a buffer of 20 mM Tris–HCl and 500 mM NaCl, at pH 7.8. The absorption spectrum scan was recorded from 250–700 nm.

### Dynamic light scattering of AgPPO8

The protein sample (7.2 mg/mL) in 20 mM Tris–HCl and 500 mM NaCl, at pH 7.8 was added into the ZMV1002 quartz batch cuvette and particle size was measured by using dynamic light scattering in the dual capability Zetasizer μV (Malvern, Inc.).

### Docking analysis

The docking of tyrosine or dopamine into the AgPPO8 active site was performed using AutoDock (version 4.2.6) [74]. The structures of tyramine and dopamine were obtained from Protein Database Bank. Based on the analysis from the previously published structures, H223, 227, 252, 379, 383, 419, L98, F99, F248, F415, E364, N380, and V406 were selected as flexible residues, and all of the bonds between CuA and CuB, except for that of V406, were inactivated, to allow slight protein dynamics upon substrate binding. Lamarckian genetic algorithm with 2,500,000 evaluations per run was chosen as the searching method. Default settings were used for all other docking parameters. The copper parameters were set as *r* (van der Waal’s radii) = 3.50, ε (vdW well depth) = 0.005 kcal/mol, and a charge of +2.0e. The docked conformation with the lowest docked energy and correct orientation (with the phenol groups of the substrate pointing towards the dicopper site) was selected for binding analysis.

### Enzyme activity assay

Enzymatic activities were measured by a microplate assay as previously described [57]. For determination of diphenol oxidase activity, 0.5 μg PPO, 50 μM CuCl_2_ and 0.002 % (w/v) CPC were mixed in the buffer of 20 mM Tris–HCl at pH 7.5, bringing a final volume to 15 μL. The mixture was incubated at room temperature for 10 min, followed by addition of 150 μL of 2 mM dopamine dissolved in 50 mM MOPS buffer at pH 6.5. The absorbance at 470 nm was monitored on a plate reader (Molecular Device VersaMax). One unit of PO activity was defined as the amount of activated PPO causing the increase of 0.001 absorbance unit per min. For hydroxylase activity measurement, 5 μg AgPPO8 was incubated with 50 μM CuCl_2_ and 0.02 % (w/v) CPC in the same way and 150 μL of 2 mM tyramine was used as the substrate. Dopamine formation was detected as the increase of absorbance at 280 nm [75]. Note that the absorbance increase at 280 nm includes a small contribution from the further dopamine–dopamine quinone conversion catalyzed by CPC-activated PPO. For each reaction, three replicates were performed and specific activity (U/μg) was presented as mean ± SEM.

### Differential scanning fluorimetry

AgPPO8 wild type and E364Q mutant proteins were purified in the buffer containing 100 mM HEPES and 150 mM NaCl, at pH 7.5; 40 μL of the protein at 0.3 mg/mL concentration were mixed with 0.8 μL of 100× SYPRO-Orange fluorescence dye (Invitrogen) to bring to a final 2× concentration. Thermal denaturation curves were monitored on Bio-Rad CFX Connect Real-time PCR Detection System, with a thermal gradient of 0.5 °C increment per 30 seconds from 24–95 °C (excitation wavelength at 515–535 nm, emission wavelength at 560–580 nm). For each reaction, three replicates were performed, and the *Tm* value was calculated using the CFX manager software v3.1 (Bio-Rad Laboratories, Inc.).

### Availability of supporting data

The atomic coordinates and structure factors of AgPPO8 have been deposited in the Protein Data Bank, www.rcsb.org (PDB accession code 4YZW).

## Additional files

Additional file 1:
***AgPPO8 exists as a homodimer in solution.*** (A) AgPPO8 appears as dimeric on size exclusion chromatography (SEC). The theoretical molecular weight (MW) of AgPPO8 monomer is 81 kDa, calculated by ExPASy server (http://web.expasy.org/compute_pi/). The retention volumes are aligned between the standard proteins (Upper panel) and AgPPO8 (Lower panel). Standards: thyroglobulin (bovine), 670 kDa; γ-globulin (bovine), 158 kDa; ovalbumin (chicken), 44 kDa; myoglobin (horse), 17 kDa; vitamin B12, 1.35 kDa. AgPPO8 was eluted out at a similar retention volume as that of γ-globulin (bovine). The calculated MW of AgPPO8 from the chromatograph is 142 kDa, indicating a dimer. (B) AgPPO8 displays as dimeric from dynamic light scattering. AgPPO8 at a concentration of 1.5 mg/mL was used in the experiment. AgPPO8 displays as monodisperse in solution with a MW of 165.9 ± 30.5 kDa, corresponding to a dimer and consistent with the result from SEC analysis. (TIF 1376 kb) Additional file 2:
***The electron density map at the*** ***di-nuclear*** ***active site of AgPPO8.*** The electron densities of 2Fo-Fc map and Fo-Fc difference map are contoured at the sigma level of 1.0 and 3.0, and shown as gray and green mesh, respectively. (TIF 3813 kb) Additional file 3:
***The six*** ***Cu-coordinating*** ***His residues are stabilized by three Phe residues at the active site of AgPPO8 via hydrophobic interactions.*** The six His and three Phe residues are shown as magenta and red sticks, respectively. The shortest carbon-carbon distances (Å) are indicated as black (F99), yellow (F248), and green (F415) dashes, respectively. CuA and CuB are shown as brown spheres. (TIF 3116 kb) Additional file 4:
***UV/Vis absorption spectrum of AgPPO8.*** The purified AgPPO8 (0.5 mg/mL) was used for absorbance scanning from 250 to 700 nm. A sharp peak at 280 nm was observed for the protein and no obvious absorbance peak was detected at other wavelength. (TIF 270 kb) Additional file 5:
***Docking of substrates to the Site II pocket of AgPPO8.*** (A) The binding energy for each individually docked conformation of tyramine/dopamine is ranked and listed in the table. (B) Superposition of all docked conformations with the correct orientations of tyramine (left) and dopamine (right). The best models of the bound substrates with the lowest binding energies are shown as yellow sticks, with all the other docking modes shown in lines. (TIF 799 kb) Additional file 6:
***Characterization of the purified AgPPO8 wild type (WT) and E364Q mutant proteins.*** (A) SDS-PAGE analysis of the purified AgPPO8 WT and E364Q mutant under reducing and non-reducing conditions. (B) Thermal denaturation (*Tm*) values (°C) of AgPPO8 WT and E364Q mutant. All data were presented as mean ± SD (n = 3). (TIF 623 kb) Additional file 7:
***Docking analysis of phenolic substrates into the*** ***F99-deleted*** ***AgPPO8 active site.*** The docked active site structures, with (magenta) or without (silver) the putative placeholder F99, are superimposed. (A) tyramine, (B) dopamine. The docked substrates in the active site are shown as yellow in F99-containing and green in the absence of F99, respectively. The envelope of Site II pocket is delineated in cyan, while CuA and CuB are shown as brown spheres. All the docking parameters were the same with the only difference being the presence or absence of F99. The best models with the lowest binding energy and correct orientation were selected for analysis. (TIF 1513 kb)
