# Supplementary figures and images for: The structure of a prophenoloxidase (PPO) from Anopheles gambiae provides new insights into the mechanism of PPO activation
Source: BMC Biol. 2016 Jan 5;14:2. doi: 10.1186/s12915-015-0225-2 (PMC4700666; doi:10.1186/s12915-015-0225-2)

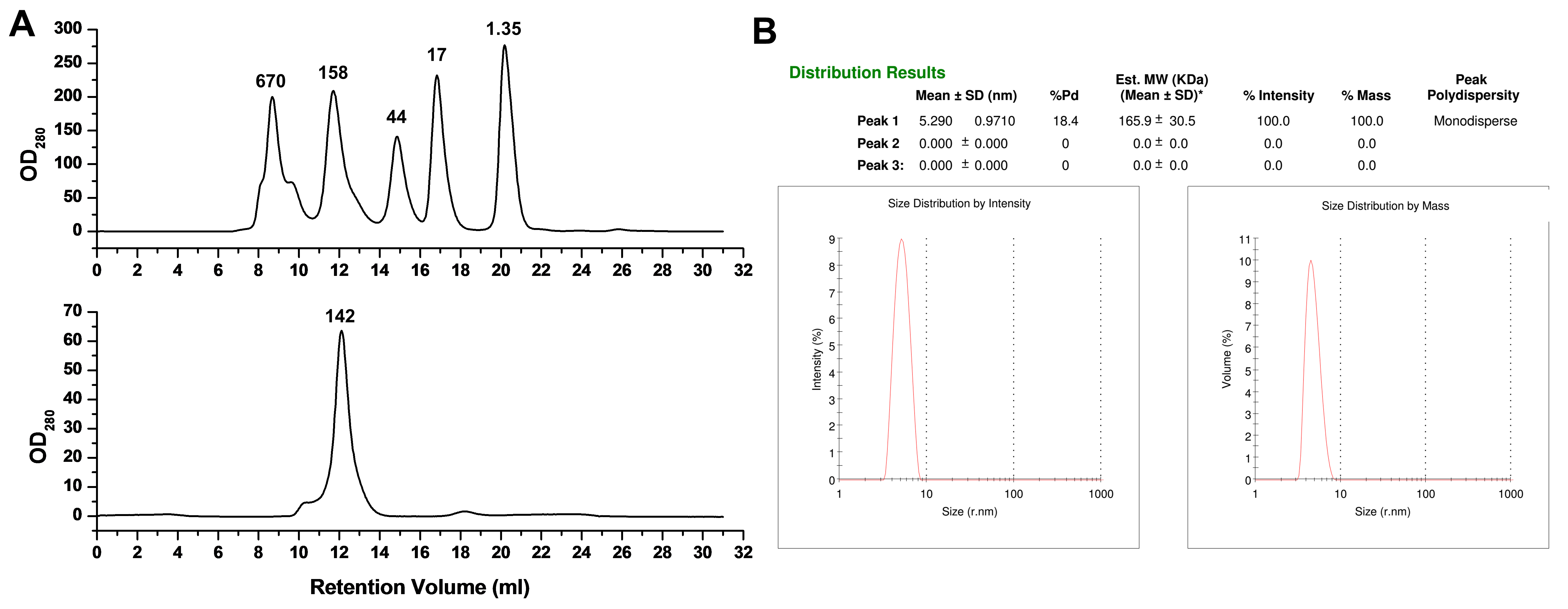

Supplement: Additional file 1: — AgPPO8 exists as a homodimer in solution. (A) AgPPO8 appears as dimeric on size exclusion chromatography (SEC). The theoretical molecular weight (MW) of AgPPO8 monomer is 81 kDa, calculated by ExPASy server (http://web.expasy.org/compute_pi/). The retention volumes are aligned between the standard proteins (Upper panel) and AgPPO8 (Lower panel). Standards: thyroglobulin (bovine), 670 kDa; γ-globulin (bovine), 158 kDa; ovalbumin (chicken), 44 kDa; myoglobin (horse), 17 kDa; vitamin B12, 1.35 kDa. AgPPO8 was eluted out at a similar retention volume as that of γ-globulin (bovine). The calculated MW of AgPPO8 from the chromatograph is 142 kDa, indicating a dimer. (B) AgPPO8 displays as dimeric from dynamic light scattering. AgPPO8 at a concentration of 1.5 mg/mL was used in the experiment. AgPPO8 displays as monodisperse in solution with a MW of 165.9 ± 30.5 kDa, corresponding to a dimer and consistent with the result from SEC analysis. (TIF 1376 kb) [file 12915_2015_225_MOESM1_ESM.tif]

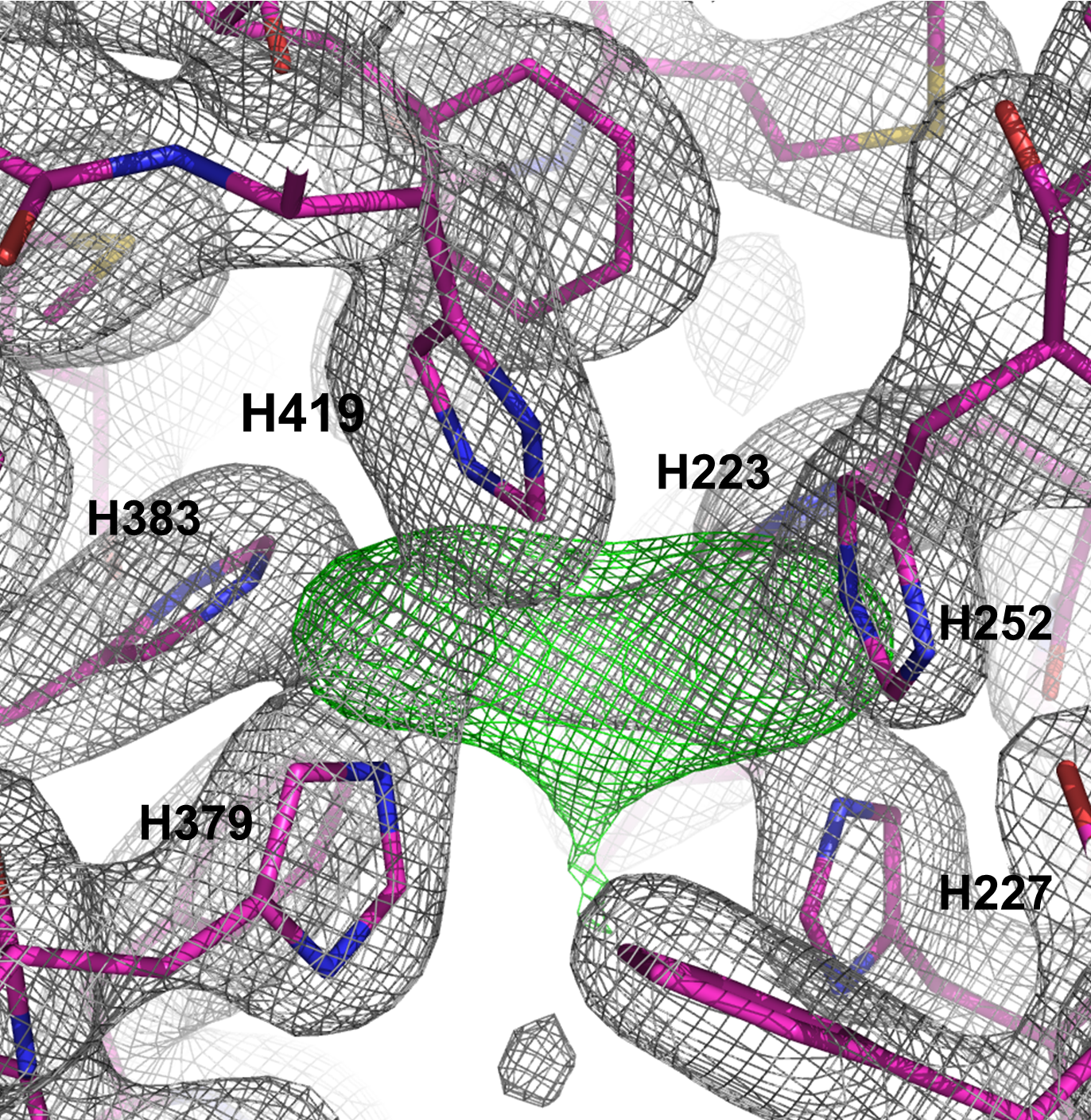

Supplement: Additional file 2: — The electron density map at the di-nuclear active site of AgPPO8. The electron densities of 2Fo-Fc map and Fo-Fc difference map are contoured at the sigma level of 1.0 and 3.0, and shown as gray and green mesh, respectively. (TIF 3813 kb) [file 12915_2015_225_MOESM2_ESM.tif]

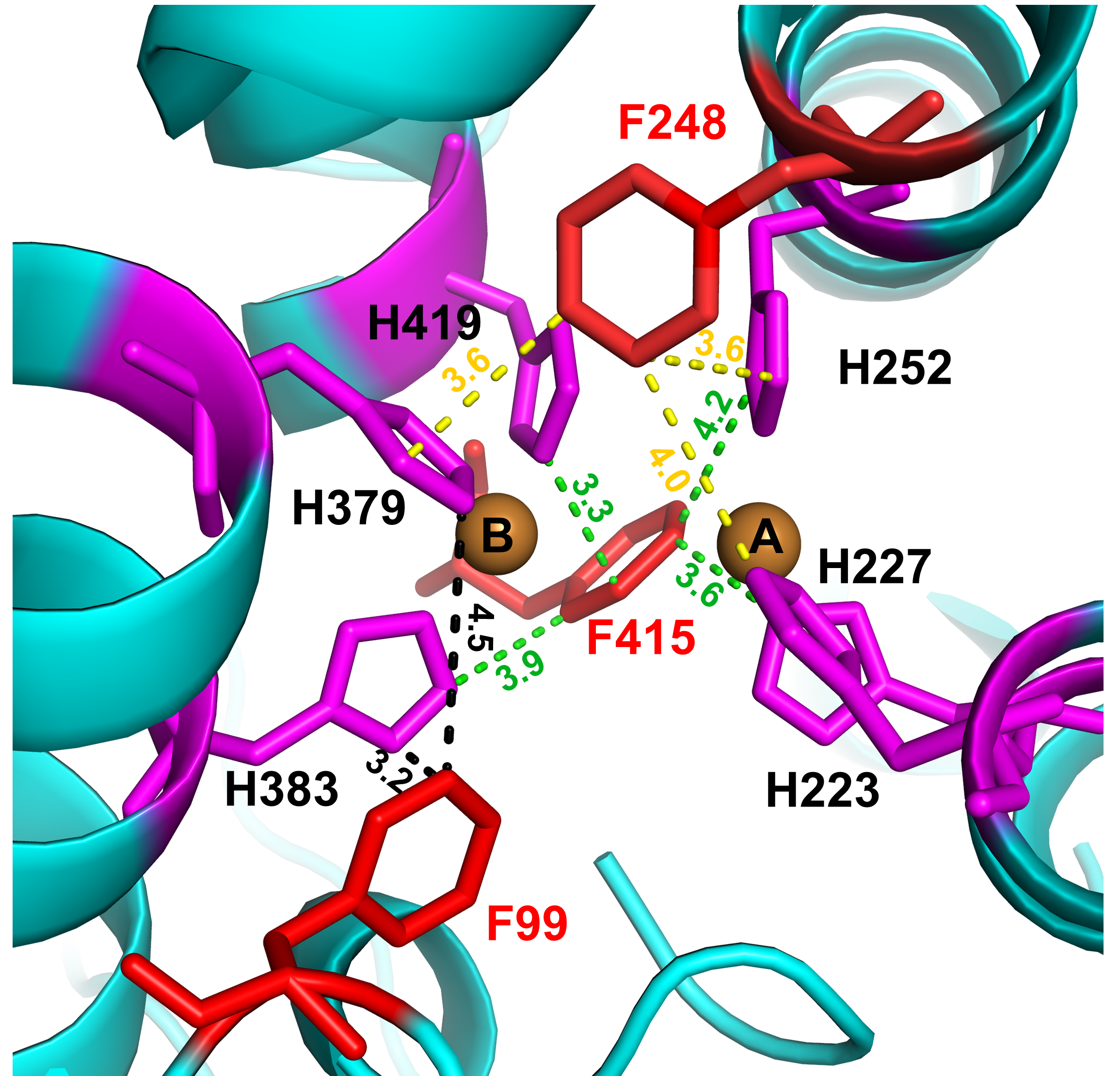

Supplement: Additional file 3: — The six Cu-coordinating His residues are stabilized by three Phe residues at the active site of AgPPO8 via hydrophobic interactions. The six His and three Phe residues are shown as magenta and red sticks, respectively. The shortest carbon-carbon distances (Å) are indicated as black (F99), yellow (F248), and green (F415) dashes, respectively. CuA and CuB are shown as brown spheres. (TIF 3116 kb) [file 12915_2015_225_MOESM3_ESM.tif]

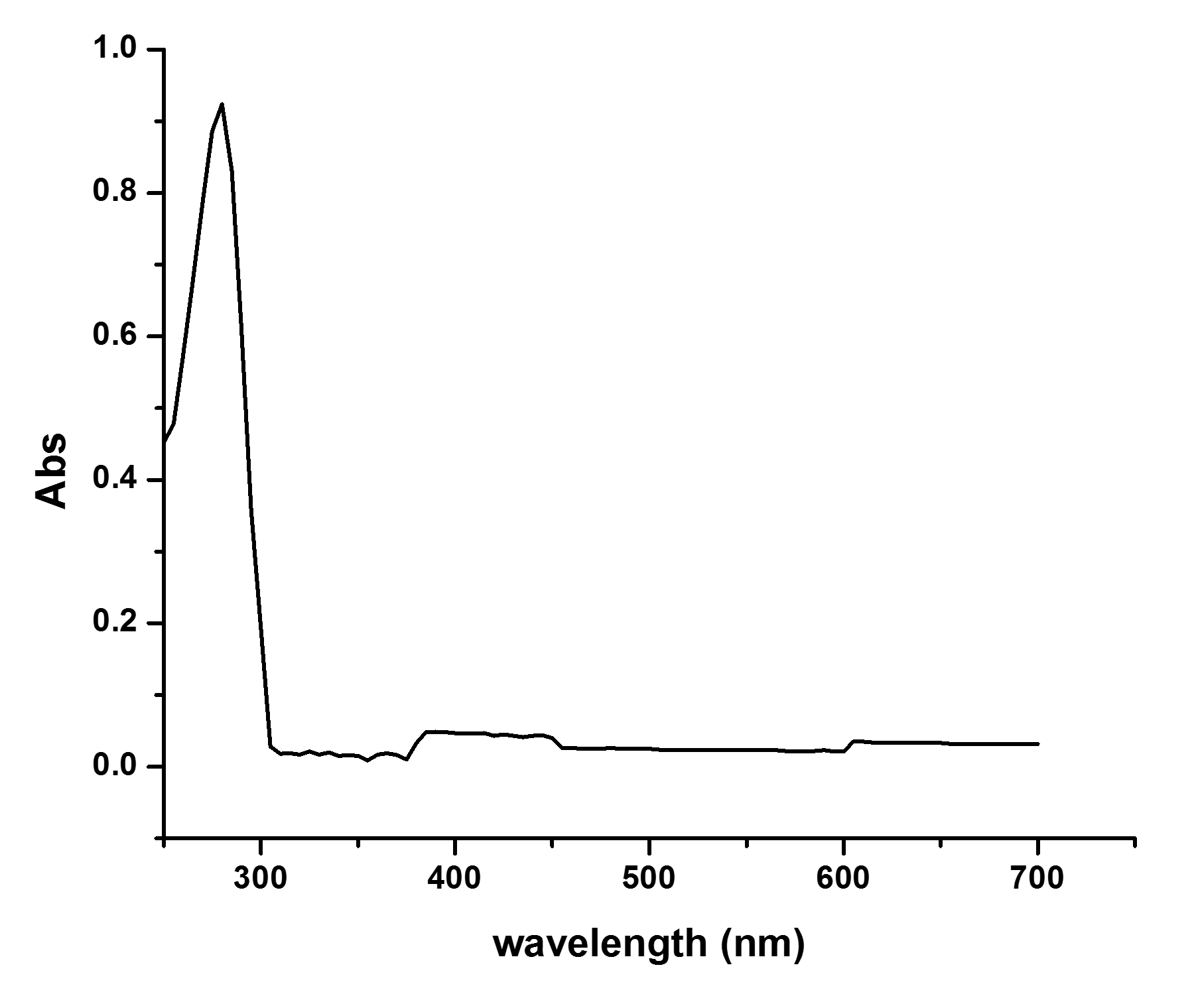

Supplement: Additional file 4: — UV/Vis absorption spectrum of AgPPO8. The purified AgPPO8 (0.5 mg/mL) was used for absorbance scanning from 250 to 700 nm. A sharp peak at 280 nm was observed for the protein and no obvious absorbance peak was detected at other wavelength. (TIF 270 kb) [file 12915_2015_225_MOESM4_ESM.tif]

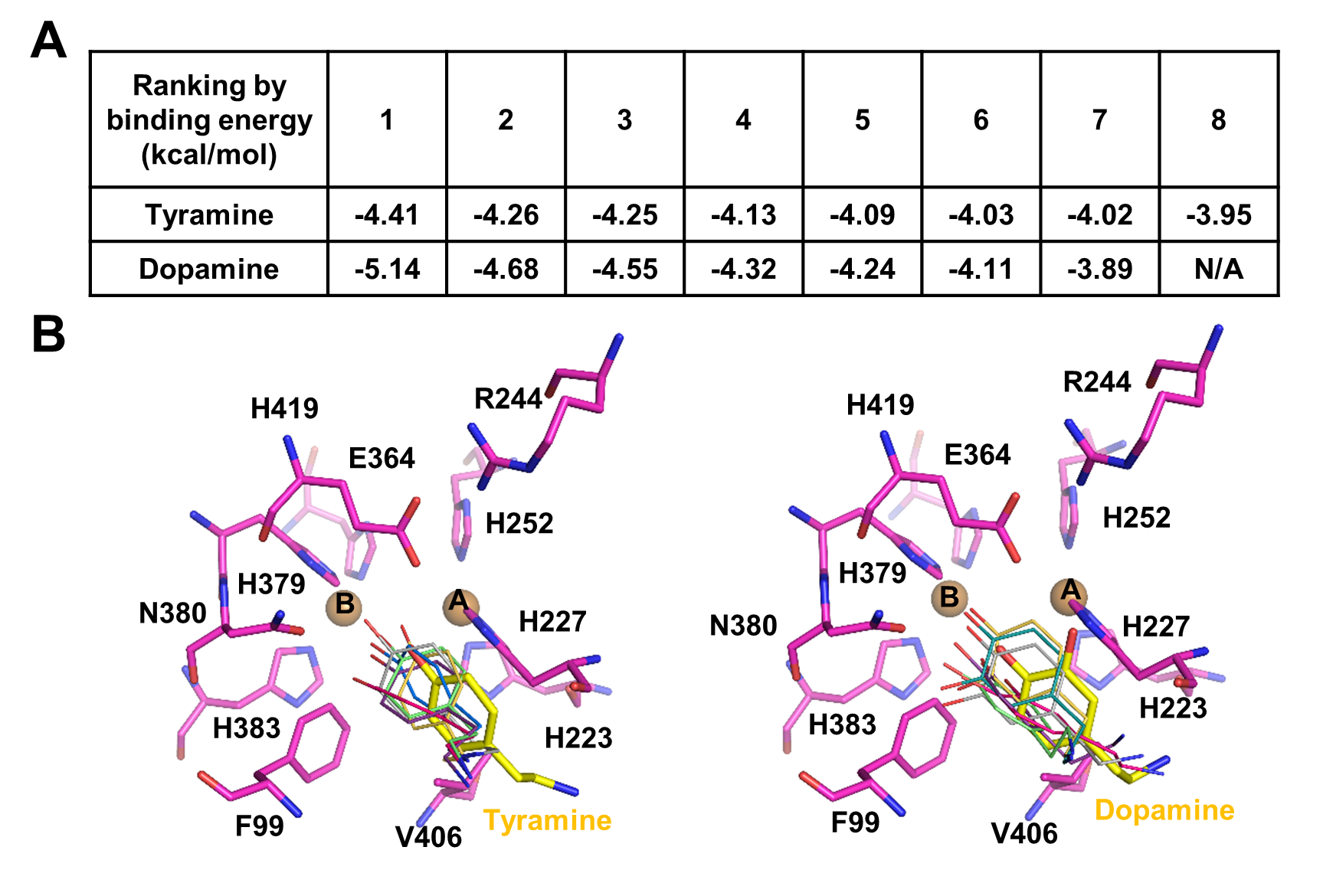

Supplement: Additional file 5: — Docking of substrates to the Site II pocket of AgPPO8. (A) The binding energy for each individually docked conformation of tyramine/dopamine is ranked and listed in the table. (B) Superposition of all docked conformations with the correct orientations of tyramine (left) and dopamine (right). The best models of the bound substrates with the lowest binding energies are shown as yellow sticks, with all the other docking modes shown in lines. (TIF 799 kb) [file 12915_2015_225_MOESM5_ESM.tif]

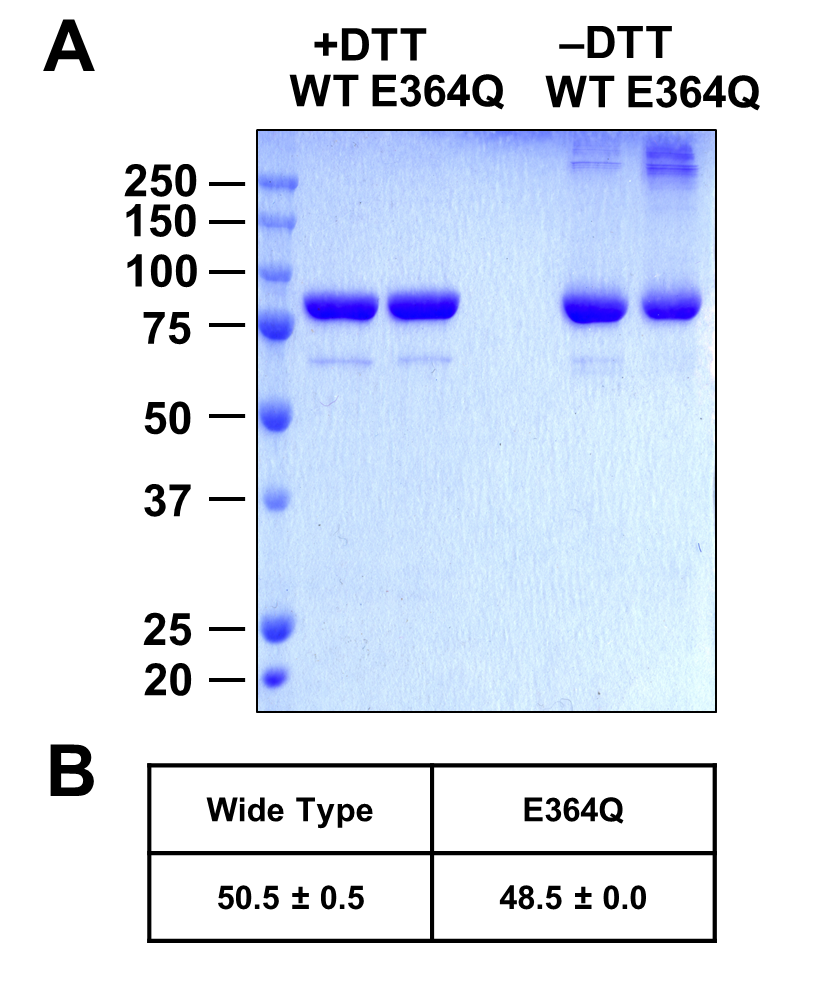

Supplement: Additional file 6: — Characterization of the purified AgPPO8 wild type (WT) and E364Q mutant proteins. (A) SDS-PAGE analysis of the purified AgPPO8 WT and E364Q mutant under reducing and non-reducing conditions. (B) Thermal denaturation (Tm) values (°C) of AgPPO8 WT and E364Q mutant. All data were presented as mean ± SD (n = 3). (TIF 623 kb) [file 12915_2015_225_MOESM6_ESM.tif]

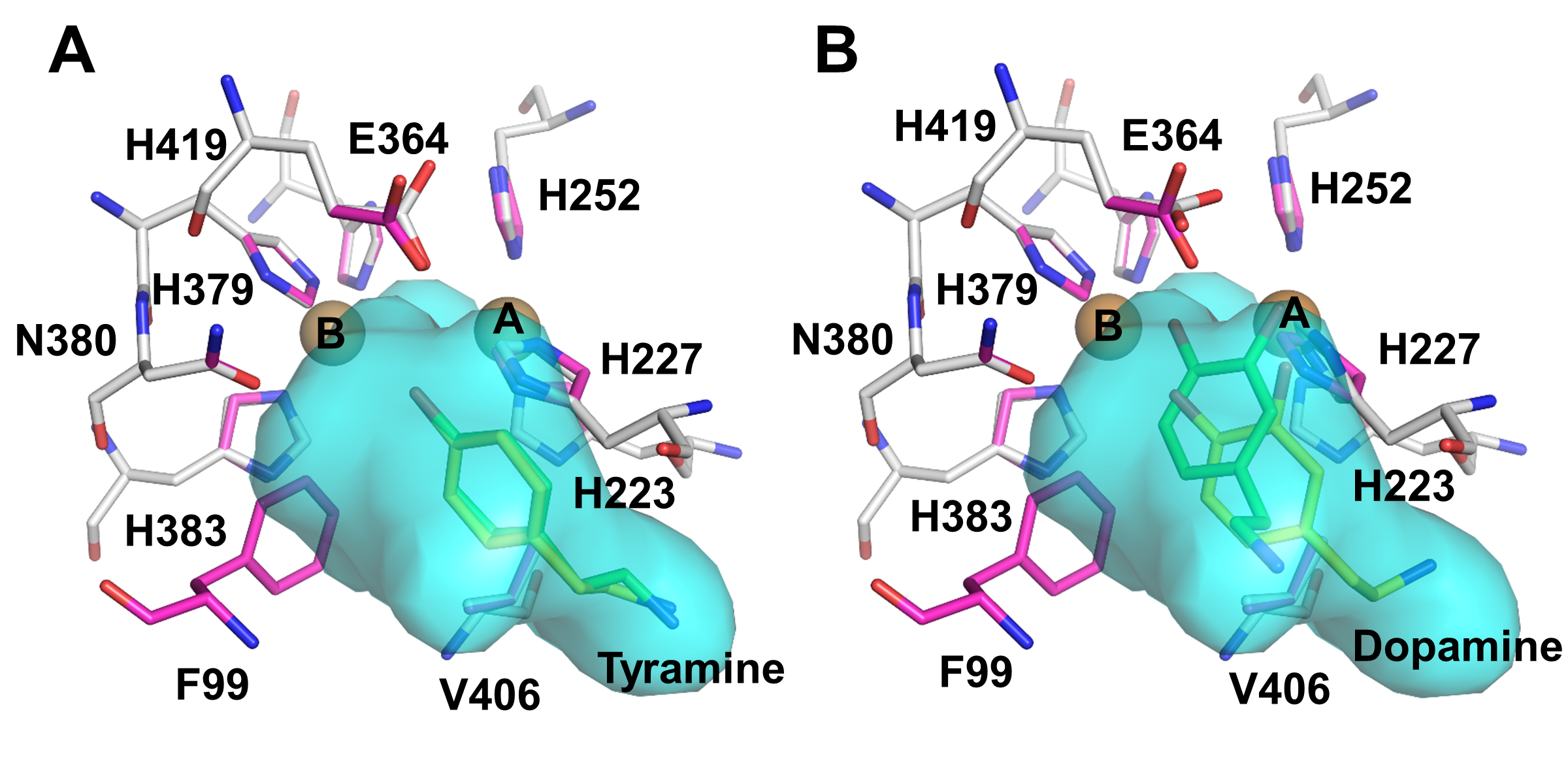

Supplement: Additional file 7: — Docking analysis of phenolic substrates into the F99-deleted AgPPO8 active site. The docked active site structures, with (magenta) or without (silver) the putative placeholder F99, are superimposed. (A) tyramine, (B) dopamine. The docked substrates in the active site are shown as yellow in F99-containing and green in the absence of F99, respectively. The envelope of Site II pocket is delineated in cyan, while CuA and CuB are shown as brown spheres. All the docking parameters were the same with the only difference being the presence or absence of F99. The best models with the lowest binding energy and correct orientation were selected for analysis. (TIF 1513 kb) [file 12915_2015_225_MOESM7_ESM.tif]
